# Association of Thyroid Function with Suicidal Behavior: A Systematic Review and Meta-Analysis

**DOI:** 10.3390/medicina57070714

**Published:** 2021-07-15

**Authors:** Freddy J. K. Toloza, Yuanjie Mao, Lakshmi Menon, Gemy George, Madhura Borikar, Soumya Thumma, Hooman Motahari, Patricia Erwin, Richard Owen, Spyridoula Maraka

**Affiliations:** 1 Division of Endocrinology and Metabolism, University of Arkansas for Medical Sciences, Little Rock, AR 72205, USA; ftolozabonilla@uams.edu (F.J.K.T.); yuanjiemao@gmail.com (Y.M.); LPMenon@uams.edu (L.M.); gemy.maria@gmail.com (G.G.); madhuborikar@gmail.com (M.B.); SPThumma@uams.edu (S.T.); HMotahari@uams.edu (H.M.); 2 Knowledge and Evaluation Research Unit in Endocrinology (KER_Endo), Mayo Clinic, Rochester, MN 55902, USA; 3 Department of Medicine, MetroWest Medical Center, Tufts Medical School, Framingham, MA 01702, USA; 4 Mayo Clinic Libraries, Mayo Clinic, Rochester, MN 55902, USA; erwin.patricia@mayo.edu; 5 Department of Psychiatry, College of Medicine, University of Arkansas for Medical Sciences, Little Rock, AR 72205, USA; Richard.Owen2@va.gov; 6 Center for Mental Healthcare and Outcomes Research, Central Arkansas Veterans Healthcare System, Little Rock, AR 72205, USA; 7 Central Arkansas Veterans Healthcare System, Medicine Service, Little Rock, AR 72205, USA

**Keywords:** suicide, thyroid function, hypothyroidism, hyperthyroidism

## Abstract

Thyroid disease is a very common condition that influences the entire human body, including cognitive function and mental health. As a result, thyroid disease has been associated with multiple neuropsychiatric conditions. However, the relationship between thyroid dysfunction and suicide is still controversial. We conducted a systematic review and meta-analysis to describe the association of thyroid function with suicidal behavior in adults. We searched four data bases (MEDLINE, EMBASE, PsycINFO, and Scopus) from their inception to 20 July 2018. Studies that reported mean values and standard deviation (SD) of thyroid hormone levels [Thyroid-stimulating hormone (TSH), free thyroxine (FT4), free triiodothyronine (FT3), total thyroxine (TT4), and total triiodothyronine (TT3)] in patients with suicidal behavior compared with controls were included in this meta-analysis. The abstracts and papers retrieved with our search strategies were reviewed independently and in duplicate by four reviewers for assessment of inclusion criteria and data extraction, as well as for evaluation of risk of bias. Random-effects models were used in this meta-analysis to establish the mean difference on thyroid function tests between groups. Overall, 2278 articles were identified, and 13 studies met the inclusion criteria. These studies involved 2807 participants, including 826 participants identified with suicidal behavior. We found that patients with suicide behavior had lower levels of FT3 (−0.20 pg/mL; *p* = 0.02) and TT4 (−0.23 µg/dL; *p* = 0.045) compared to controls. We found no differences in either TSH, FT4, or TT3 levels among groups. With our search strategy, we did not identify studies with a comparison of overt/subclinical thyroid disease prevalence between patients with and without suicide behavior. The studies included in this meta-analysis had a low-to-moderate risk of bias. In the available literature, the evidence regarding the association of thyroid disorders and suicidal behavior is limited. We found that patients with suicidal behavior have significantly lower mean FT3 and TT4 levels when compared to patients without suicidal behavior. The clinical implications and pathophysiologic mechanisms of these differences remain unknown and further research is needed.

## 1. Introduction

Suicide is one of the main causes of mortality around the globe, accounting for about 800,000 deaths every year [1]. Deaths by suicide are more frequent in individuals with psychiatric disorders, such as major depression and alcohol use disorder. By far the single most important risk factor for suicide is a prior suicide attempt [2]. Therefore, suicide and suicide attempt/ideation are serious public health problems, as these disorders deeply affect families and communities and have long-lasting effects on the people left behind.

Thyroid dysfunction is the most common endocrine disorder [3]. Most current estimates showed that around 12% of Americans will develop a thyroid condition during their lifetime and that around 20 million have a thyroid disorder [4]. Additionally, it has been estimated that one in eight women will develop a thyroid disorder during their lifetime [5]. Some endocrine disorders have been correlated with psychiatric conditions, such as major depression, anxiety, etc. [6,7,8,9]. This is not the case for suicidal behavior, as its relationship with thyroid dysfunction is controversial, and mostly based on observational studies.

A nationwide Danish register-based study, including 111,565 participants, reported that mortality due to suicide was increased (0.10% vs. 0.07%, *p* < 0.001) in patients with Hashimoto’s thyroiditis compared to matched controls, suggesting a possible role of Hashimoto’s thyroiditis in the pathophysiologic mechanisms of suicidal behavior [10]. Similarly, a study including 1718 patients with a history of major depressive disorder showed that those with history of suicide attempt had higher levels of thyroid-stimulating hormones (TSH) and thyroid autoantibodies compared with those without history of suicide attempt, suggesting a potential role of alterations in thyroid function tests in the risk of suicide attempts [11]. In contrast, in a large prospective cohort study of women with thyroid dysfunction that explored the risk of cause-specific mortality after nearly 30 years of follow-up, neither hyperthyroidism nor hypothyroidism were associated with higher rates of suicide [12].

Due to the scarcity of evidence and the conflicting findings among observational studies, we conducted, to our knowledge, the first systematic review and meta-analysis exploring the association between thyroid dysfunction with suicidal behavior in adults.

## 2. Materials and Methods

### 2.1. Search Strategy

We conducted a comprehensive search in four data bases (Ovid MEDLINE Epub Ahead of Print, Ovid Medline In-Process & Other Non-Indexed Citations, Ovid MEDLINE, Ovid EMBASE, Ovid, PsycINFO, and Scopus) from their inception to 20 July 2018, with no language restrictions. Our search strategy was designed and conducted by an experienced librarian (P.E.J.) with input from the principal investigator (S.M.) (File S1).

A systematic review software (DistillerSR, Ottawa, ON, Canada) was used to upload the results of the search strategies, for title and abstracts screening, and for full-text review. Each paper was reviewed by two reviewers working in an independent manner. First, we screened abstracts for eligibility, and those that could be potentially included in our meta-analysis underwent an in-depth review of the full-text paper. The chance-adjusted inter-reviewer agreement among reviewers was high (kappa statistic = 0.82). Any disagreement during the reviewing process was resolved by the principal investigator (S.M.).

### 2.2. Study Selection

For this systematic review and meta-analysis, studies reporting mean values of thyroid function tests [TSH, free thyroxine (FT4), free triiodothyronine (FT3), total thyroxine (TT4), and total triiodothyronine (TT3)] along with their standard deviation (SD) in patients with suicidal behavior (recent episode or history of suicidal ideation/suicide attempt) compared with patients without suicidal behavior were included. We contacted the authors of those papers in which additional information or clarification was required in order to confirm eligibility. In cases where we were not able to reach them, these studies were excluded from the analyses. Studies with only abstracts available, papers published in languages other than English or Spanish, papers without a quantitative assessment of thyroid function, and papers without a comparison to a control group (group without suicidal behavior) were excluded.

### 2.3. Data Extraction and Quality Assessment

We designed and used a standardized form to retrieve data from each study included in the meta-analysis. The data extracted included: first author name, paper title, publication year, country, publishing journal, study design, type and criteria used to define the group with suicidal behavior and control group, sample size of suicidal behavior group and control group, mean and SD of age by groups, percentage of female by groups, and mean and SD of thyroid function tests (TSH, FT4, TT4, FT3, TT3) by groups. The data from each paper were extracted independently and in duplicate by four reviewers.

For the assessment of methodological quality and risk of bias of the included studies we used the Newcastle-Ottawa risk of bias tool for observational studies [13]. This tool has been extensively validated and is used to assess the comparability of the study groups, their representativeness, and the ascertainment of exposure and outcomes. Reviewers worked independently, assessing the risk of bias of the included studies. Any disagreement during this stage was resolved by consensus.

### 2.4. Statistical Analysis

The main outcomes summarized in this meta-analysis were the mean difference (MD) in thyroid function tests for the suicidal behavior group compared with the control group. We calculated and pooled unstandardized MDs of the included studies using random effects models [14]. In the case of multiple subgroups from one study sharing the same comparison group, these groups were combined following the recommended procedure [14]. We used the I^2^ statistic to estimate the percentage of total between-study variation due to heterogeneity rather than chance [14]. Sensitivity analyses, when possible, were performed according to the type of suicidal behavior of the study population (recent suicide attempt, recent suicidal ideation, history of suicidal attempt, history of suicidal ideation). We performed leave-one-out sensitivity analyses by iteratively removing one study at a time and recalculating the summary MD for those outcomes assessed in ≥10 studies. We assessed publication bias with funnel plot for visual detection of asymmetries and Egger’s tests for the detection of statistical asymmetry in the funnel plot when ≥10 studies were available for a specific outcome [15,16]. All values are two-tailed, and *p* < 0.05 was set as the threshold for statistical significance. Review Manager v5.4, OpenMeta[Analyst], and IBM SPSS statistics v26 were used for statistical analyses.

## 3. Results

### 3.1. Study Selection and Characteristics

**Figure 1:**
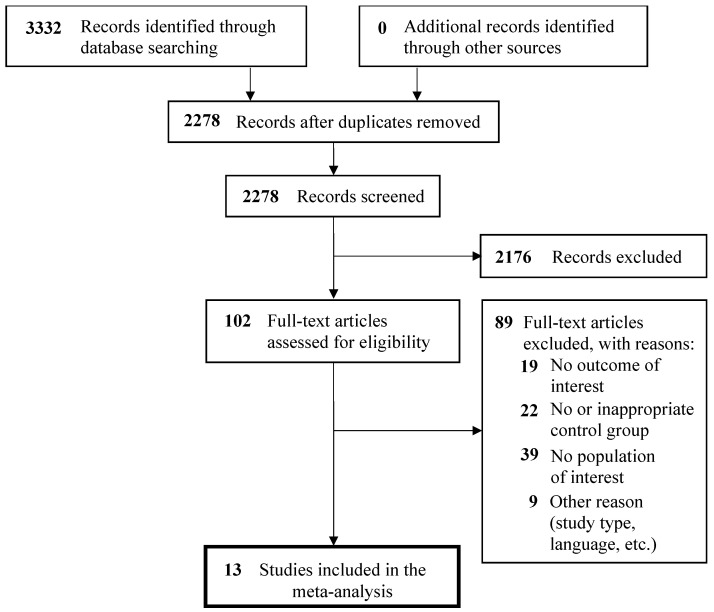
PRISMA flow diagram.

Our search strategies identified 2278 articles, of which 102 were evaluated as full text and 13 studies [17,18,19,20,21,22,23,24,25,26,27,28,29] were finally selected for the meta-analysis (Figure 1). Table 1 summarizes the characteristics of the studies included in the meta-analysis. These studies involved 2807 participants, of whom 826 participants had suicidal behavior. Sample sizes ranged from 20 to 555 participants; mean ages of the participants ranged from 23 to 50 years. All the included studies reported thyroid function tests as mean and SD for the suicidal behavior group compared with control group. The studies included in this meta-analysis have a low-to-moderate risk of bias (Supplementary Table S1).

### 3.2. Suicidal Behavior and Thyroid Function Tests

**Figure 2:**
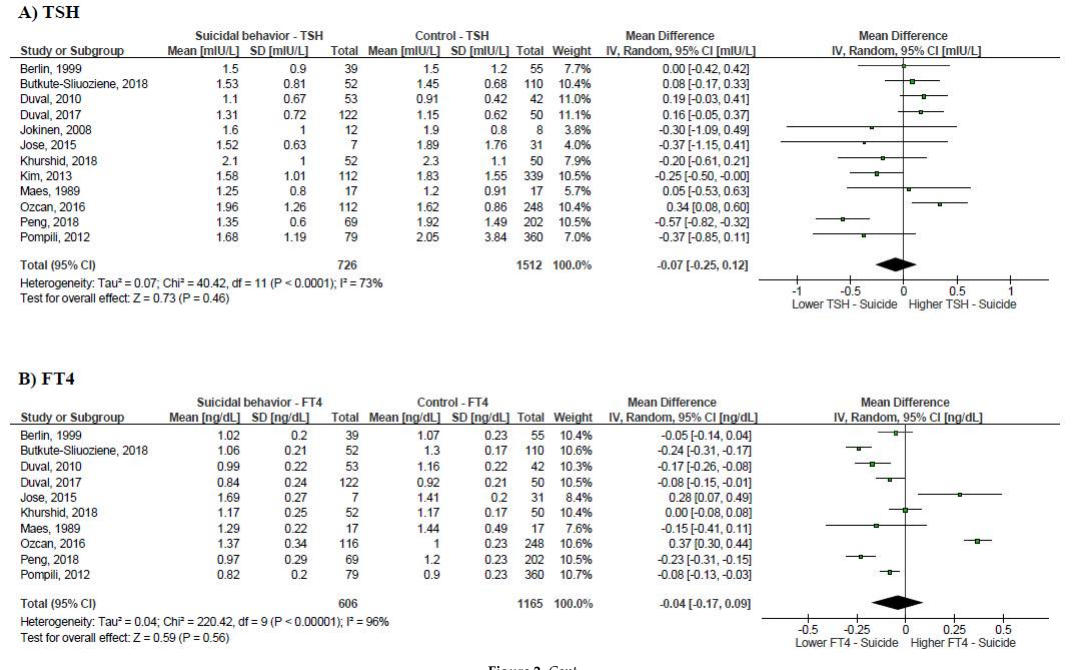

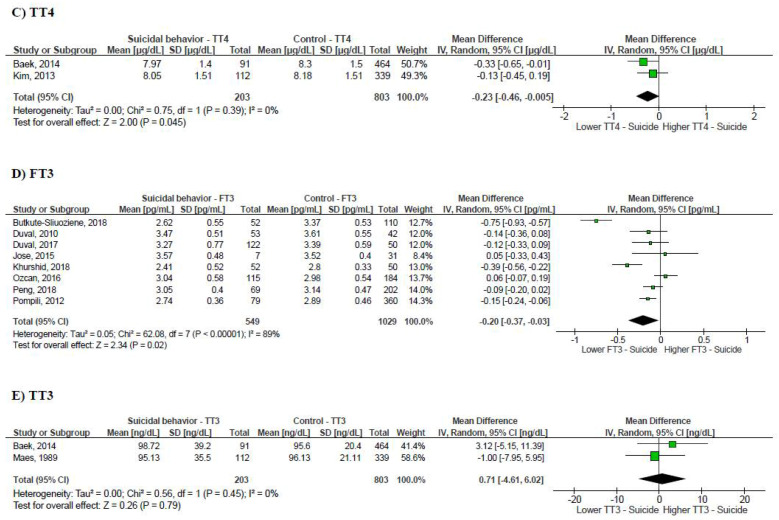
Forest plots summarizing the mean difference of TSH, FT4, TT4, FT3, and TT3 levels in patients with suicidal behavior compared with control group.

We found that patients with suicidal behavior had significantly lower levels of FT3 (−0.20 pg/mL; 95% CI, −0.37 to −0.03; *p* = 0.02; I2 = 89%) and TT4 (−0.23 µg/dL; 95% CI, −0.46 to −0.005 µg/dL; *p* = 0.045; I2 = 0%) compared to controls (Figure 2). There were no differences in the levels of TSH (−0.07 mIU/L; 95% CI, −0.25 to 0.12; *p* = 0.46; I2 = 73%), FT4 (−0.04 ng/dL; 95% CI, −0.17 to 0.09; *p* = 0.56; I2 = 96%) and TT3 (0.71 ng/dL; 95% CI, −4.61 to 6.02; *p* = 0.79; I2 = 0%) (Figure 2). We did not identify studies with a comparison of overt/subclinical thyroid disease prevalence in patients with and without suicide behavior.

In sensitivity analyses according to the type of reported suicidal behavior, we found that, similarly to the overall analysis, significantly lower levels of FT3 were maintained in patients with history of suicide attempt, who also had significantly lower levels of FT4 and higher levels of TSH (Table 2). Contrastingly, significantly lower levels of TSH were found in the group of patients with recent suicide ideation (Table 2).

### 3.3. Publication Bias and Leave-One-Out Sensitivity Analysis

We did not find major asymmetries in the funnel plots to suggest high risk of publication bias (Supplementary Figure S1). For outcomes assessed in ≥10 studies we performed Egger’s tests, TSH (*p* = 0.43) and FT4 (*p* = 0.69), which suggested no significant publication bias existed for these outcomes. We performed a leave-one-out sensitivity for those outcomes assessed in ≥10 studies (TSH and FT4 levels) and the summary MDs remained stable (Supplementary Figure S2). This finding indicates that our results were not driven by any single study and that similar results could be obtained after excluding individual studies.

## 4. Discussion

In this systematic review and meta-analysis, we found that patients with suicidal behavior have lower levels of FT3 and TT4 compared with controls, but we found no difference in TSH, FT4, or TT3 levels. Furthermore, the subgroup of patients with history of suicide attempt had lower FT3 and FT4 levels, and higher TSH levels compared to controls. This study represents the first summary about the relationship between suicidal behavior with thyroid function.

Our results are in line with previously published evidence regarding the risk/association of thyroid disorders with suicide behavior [10,30,31]. Heiberg-Brix et al. found that patients with Hashimoto’s thyroiditis had an increased frequency of death by suicide (HR = 1.31; 95% CI, 1.04–1.65, *p* = 0.024) compared to euthyroid controls in a register-based Danish study [10]. In contrast, this frequency is not increased in patients with Graves’ disease compared to controls without Graves’ disease or euthyroid controls from the general population [32,33]. According to these findings, suicide possibly has a stronger association with autoimmune hypothyroidism rather than autoimmune hyperthyroidism. Similar results have been reported for suicidal attempt/ideation and thyroid disorders. A cross-sectional study showed that the prevalence of hypothyroidism in a group of 31 patients with bipolar disorder and suicide attempt was higher (25.8%) compared with a prevalence of 15.9% in a group of 63 patients with bipolar disorder with no suicide attempt [30]. Sanna et al. reported a 6.5% prevalence of thyroid disorders in males with history of suicidal ideation, compared with a 1.9% prevalence in males without history of suicidal ideation [31]. Nevertheless, these results should be analyzed carefully as all these studies are observational with relatively small sample size and analyses are not adjusted for confounders.

The development of suicide, depression, and other affective disorders has been previously associated with autoimmune diseases, including thyroid-specific diseases [10,34,35,36]. In a meta-analysis published by Siegmann et al., patients with Hashimoto thyroiditis and either subclinical or overt hypothyroidism showed significantly higher scores on standardized depression instruments compared to euthyroid controls [37]. A second meta-analysis published in 2019, found that patients with subclinical hypothyroidism had higher risk of depression than euthyroid controls. However, there was no difference in the mean TSH level between individuals with depression and healthy controls [38]. Although the majority of patients with depression and other mood disorders have completely normal thyroid function tests, several test abnormalities have been described including elevated TT4 levels, low TT3 and FT3 levels, blunted TSH response to thyrotropin-releasing hormone, and positive antithyroid antibodies [36,39,40,41,42,43,44,45]. Interestingly, thyroid hormone in the form of liothyronine has been used for the treatment of depression, mainly as an augmentation therapy in severe forms of depression [46,47,48,49]. In addition, in the setting of suicidal behavior, there is evidence suggesting that thyroid hormones might have a role in the regulation of the neurotransmitters involved in suicide pathogenesis, such as serotonin and norepinephrine [50,51,52,53]. As demonstrated by our results, where most of the participants had concomitant affective disorders (depression or bipolar disorder) or schizophrenia, patients with suicidal behavior had significantly lower levels of FT3 and TT4, albeit not clinically meaningful, when compared with the control group.

To our knowledge, this is the first systematic review and meta-analysis of available evidence evaluating the relationship between suicide and thyroid function tests. There were some limitations to our study. First, due to the observational nature of all the studies included in this meta-analysis, it was not possible to establish causality. Second, although we used clear and standardized inclusion criteria and comprehensive search strategies, there remains possible sources of bias such as incomplete searching, publication bias, and the influence of confounding factors (current therapies, psychiatric comorbidities, environmental factors, etc.) in our results. Third, there was heterogeneity of the criteria used to define suicide behavior in the included studies (based on clinical records, psychiatric assessment, self-reported, etc.) and the presence of other concomitant psychiatric diseases which can affect the risk of suicidal behavior. Importantly, suicide is a very complex phenomenon related to many mental health diagnoses and with a multitude of biological, psychological, and social variables which should be considered when interpreting our findings.

## 5. Conclusions

In conclusion, we found that the available evidence regarding the association of hypothyroidism or hyperthyroidism with suicidal behavior is limited. In our best effort to summarize all the available evidence, we have described that patients with suicidal behavior had significantly lower levels of FT3 and TT4 levels compared with controls. However, the data in this field are scarce and have significant heterogeneity. Future large, well conducted studies are needed to increase our confidence in the findings presented here, especially studies reporting the specific association of hypothyroidism/hyperthyroidism in this population, which can provide a better understanding, evaluation, and follow-up of patients with thyroid dysfunction and suicidal behavior.

## Figures and Tables

**Table 1:** Characteristics of the included study groups.

First Author	Publication Year	Country	Study Type	Suicidal Behavior Group					Control Group			
				*N*	Female (%)	Mean Age (SD)	Group Description	Suicidal Behavior Assessment	*N*	Female (%)	Mean Age (SD)	Group Description
Baek	2014	South Korea	Cross-sectional	91	78.0	40.1 (NR)	Patients newly diagnosed with major depressive disorder [Diagnostic and Statistical Manual of Mental Disorders (DSM)-IV criteria]-Recent and lifetime suicide attempters	The Korean version of the Mini International Neuropsychiatric Interview (MINI) suicidality module. Three groups: recent suicide attempters (within the last month), lifetime attempters (those who have attempted suicide in their lifetime except for the past month) and never attempters	464	72	47.5 (15.6)	Patients newly diagnosed with major depressive disorder (DSM-IV criteria)-Never suicide attempters
Berlin	1999	France	Cross-sectional	39	NR	NR	Patients with major depressive disorder (DSM-III-R criteria) and necessitating hospitalization-Previous suicide attempt	NR	55	NR	NR	Patients with major depressive disorder (DSM-III-R criteria) and necessitating hospitalization-No previous suicide attempt
Butkute-Sliuoziene	2018	Lithuania	Cross-sectional	56	66.1	36.5 (13.1)	Adult non-psychotic patients, without organic brain disorder, hospitalized due to high suicide risk (Suicide ideation or attempt)	An authors’ composed socio-demographic questionnaire including questions about history and type of suicidal ideations or behavior. The majority of patients presented after a suicidal attempt (83.9%) and a small percentage with severe suicidal ideations and high risk of suicide (16.1%)	120	42.5	34.3 (13.0)	Healthy volunteers (without a history of mental disorders or suicide attempts)
Duval	2010	France	Case/Control	53	58.5	38.1 (10.7)	Inpatients with current major depressive episode (DSM-IV criteria) and recent suicidal attempt or history of suicidal attempt	Semi-structured interview with an experienced psychiatrist and a review of medical records. Recent suicide attempters: if the suicidal act occurred during the current depressive episode. Past suicide attempters, if the most recent suicide attempt had not occurred during the current depressive episode	42	45.2	40.9 (11.1)	Inpatients with current major depressive episode (DSM-IV criteria) and no history of suicidal attempt
Duval	2017	France	Case/Control	122	68	39.4 (11.8)	Adults with major depressive episode (DSM-5 criteria) (95 with major depressive disorder; 18 with bipolar I disorder, and 9 with bipolar II disorder) with a suicide attempt that occurred within the last twp years	Semi-structured interview with an experienced psychiatrist. Current/recent suicide attempt (occurred within the last year) and in early remission/history of suicide attempt (the last suicide attempt occurred 12–24 months prior to evaluation)	50	62	40.2 (8.3)	Hospitalized volunteers free of concomitant psychiatric and medical illness
Jokinen	2008	Sweden	Case/Control	12	0	32 (7.2)	Patients not receiving any antidepressant treatment admitted to the hospital after a suicide attempt	The Suicide Intent Scale performed by a psychiatrist within 48 h after admission. This 15-item instrument was designed to measure the factual aspects of a suicide attempt	8	0	24 (1.8)	Healthy volunteers without psychiatric history
Jose	2015	India	Case/Control	7	0	23.3 (3.8)	Male patients with schizophrenia (DSM IV TR criteria) aged 18 to 45 years and who were not on any treatment for schizophrenia for at least 4 weeks-Suicidal ideation present in the last three months	The Columbia Suicide Severity Rating Scale assessing suicidal ideations and behaviors for recent past (three months) was used	31	0	30.3 (7.1)	Male patients with schizophrenia (DSM IV TR criteria) aged 18 to 45 years and who were not on any treatment for schizophrenia for at least four weeks-Suicidal ideation absent in the last three months
Khurshid	2018	Pakistan	Cross-sectional	54	39	30.5 (10.1)	Consecutive patients with past history of suicide attempt/ideation	NR	50	44	NR	Psychiatric patients without suicide attempt or ideation
Kim	2013	South Korea	Cross-sectional	112	100	41.7 (13.9)	Female patients with major depressive disorder from the outpatient clinic-Suicide attempt/ideation within the last month	The Korean version of the MINI suicidality module assessing suicidal behavior within the last month	339	100	49.1 (14.7)	Female patients with major depressive disorder from the outpatient clinic-No history of suicide attempt/ideation
Maes	1989	Belgium	Cross-sectional	17	100	48.5 (10.5)	Female patients with major depressive disorder and suicidal ideation	Suicidal ideation was determined when the Hamilton Rating Scale for Depression score on item 3 (suicide) was three, and when the item on suicide of the Structured Clinical Interview for DSM-III (depression section) was definitely positive	17	100	50.4 (10.0)	Female patients with major depressive disorder with no suicide ideation
Ozcan	2016	Turkey	Case/Control	115	67.8	25.9 (11.6)	Suicide attempters who were admitted consecutively to the Emergency Room	Evaluation of DSM IV-TR criteria and details of previous and current psychiatric history within the last six months were recorded by means of a semi-structured interview by a senior and experienced psychiatry resident	243	68.4a	26.4 (6.6)a	Subjects who were admitted consecutively to the hospital with no suicide attempt
Peng	2018	China	Cross-sectional	69	59.9	36.4 (15.5)	Suicidal attempters incorporated after a suicide attempt at a university hospital	The Hamilton Depression Rating Scale and the Beck Scale for Suicidal Ideation	202	61.4	36.4 (15.7)	Subjects with a major depressive episode without suicidal behavior
Pompili	2012	Italy	Cross-sectional	79	63.3	40.8 (13.9)	Patients suffering from mood disorders and psychosis consecutively admitted to the emergency department-Suicide attempt	An interview a psychiatrist performing mental examinations relied on the MINI and one section of this instrument dedicated to the assessment of suicidal risk, with questions about past and current suicidality	360	50.6	41.8 (13.3)	Patients suffering from mood disorders and psychosis consecutively admitted to the emergency department-No suicide attempt

**Table 2:** Sensitivity analysis by type of suicidal behavior.

Sensitivity Analyses		MD [95% CI]	I2 (%)	References Included
Recent suicide attempt				
	TSH (mIU/L)	−0.03 [−0.28 to 0.22]	72	[19,20,21,23,25,27,28,29]
	FT4 (ng/dL)	−0.08 [−0.26 to 0.11]	98	[19,20,21,27,28,29]
	TT4 (µg/dL)	−0.35 [−0.11 to 0.82]	0	[17,25]
	FT3 (pg/mL)	−0.19 [−0.39 to 0.01]	91	[19,20,21,27,28,29]
	TT3 (ng/dL)	−1.41 [−2.73 to 5.54]	0	[17,25]
Recent suicidal ideation				
	TSH (mIU/L)	−0.30 [−0.51 to −0.09]	0	[22,25,26]
	FT4 (ng/dL)	−0.07 [−0.35 to 0.49]	84	[22,26]
History of suicide attempt				
	TSH (mIU/L)	0.18 [0.01 to 0.35]	0	[18,20,21,24]
	FT4 (ng/dL)	−0.06 [−0.10 to −0.01]	0	[18,20,21,24]
	FT3 (pg/mL)	−0.27 [−0.41 to −0.12]	22	[20,21,24]

MD, mean difference; CI, 95% confidence interval; TSH, thyroid stimulating hormone; FT4, free T4; FT3, free T3; TT4, total T4; TT3, total T3.

## Data Availability

Data used in this project are available upon request to contact author.

## Supplementary Materials

The following are available online at https://www.mdpi.com/article/10.3390/medicina57070714/s1. Figure S1, Funnel plots for publication bias analysis; Figure S2, Leave-one-out sensitivity analysis of the studies included; Table S1, Summary of risk of bias assessment for the included studies. File S1: Search Strategies Used in this Project.
