# Supplementary material for: Association of Thyroid Function with Suicidal Behavior: A Systematic Review and Meta-Analysis
Source: Medicina (Kaunas). 2021 Jul 15;57(7):714. doi: 10.3390/medicina57070714 (PMC8303342; doi:10.3390/medicina57070714)
Supplement: Supplementary file 1 [file medicina-57-00714-s001.zip › File S1.pdf]

Ovid MEDLINE(R) and Epub Ahead of Print, In-Process & Other Non-Indexed Citations, and Daily <1946 to July 20, 2018>

Search history sorted by search number ascending

#	Searches	Results	Type
1	ptsd.mp. or exp Stress Disorders, Post-Traumatic/	34136	Advanced
2	((posttraumatic or "post traumatic") adj2 (neurosis or stress)).mp. [mp=title, abstract, original title, name of substance word, subject heading word, floating sub-heading word, keyword heading word, protocol supplementary concept word, rare disease supplementary concept word, unique identifier, synonyms]	38058	Advanced
3	stress disorder, traumatic/ or stress, psychological/ or (psychological adj3 trauma*).mp.	111580	Advanced
4	or/1-3trau	146293	Advanced
5	exp Hypothyroidism/ or Hypothyroidism/ or hypothyroid*.mp. or hyperthyroid*.mp or thyroid*.mp. [mp=title, abstract, original title, name of substance word, subject heading word, floating sub-heading word, keyword heading word, protocol supplementary concept word, rare disease supplementary concept word, unique identifier, synonyms]	225553	Advanced
6	exp Thyroid Hormones/an, bl, df, tu [Analysis, Blood, Deficiency, Therapeutic Use]	33322	Advanced
7	(tsh or t3 or triiodothyronine or t4 or thyroxine or levothyroxin*).mp. [mp=title, abstract, original title, name of substance word, subject heading word, floating sub-heading word, keyword heading word, protocol supplementary concept word, rare disease supplementary concept word, unique identifier, synonyms]	120668	Advanced
8	5 or 6 or 7	289355	Advanced
9	4 and 8	763	Advanced

10	8 and suicid*.mp. [mp=title, abstract, original title, name of substance word, subject heading word, floating sub-heading word, keyword heading word, protocol supplementary concept word, rare disease supplementary concept word, unique identifier, synonyms]	372	Advanced
11	9 or 10	1126	Advanced
12	remove duplicates from 11	1126	

CENTRAL = 104

Embase <1988 to 2018 Week 30>

Search history sorted by search number ascending

#	Searches	Results	Type
1	exp posttraumatic stress disorder/	50249	Advanced
2	((psychological or trauma* or post trauma* or posttrauma*) adj3 (stress* or neurosis)).mp. [mp=title, abstract, heading word, drug trade name, original title, device manufacturer, drug manufacturer, device trade name, keyword, floating subheading word, candidate term word]	70812	Advanced
3	pstd.mp.	105	Advanced
4	or/1-3	70843	Advanced
5	exp hypothyroidism/	49222	Advanced
6	thyroid hormone/	24199	Advanced
7	exp thyromimetic agent/	72931	Advanced
8	exp hyperthyroidism/	30920	Advanced

9	or/5-8	123453	Advanced
10	4 and 9	246	Advanced
11	limit 10 to human	222	Advanced
12	remove duplicates from 11	219	Advanced
13	12 not case report/	184	Advanced
14	suicide attempt/ or suicidal ideation/ or suicidal behavior/ or suicide/	79342	Advanced
15	9 and 14	586	Advanced
16	limit 15 to human	572	Advanced
17	16 not case report/	410	Advanced
18	remove duplicates from 17	408	Advanced
19	13 or 18	582	

PsycINFO <1967 to July Week 3 2018>

Search history sorted by search number ascending

#	Searches	Results	Type
1	posttraumatic stress disorder/ or acute stress disorder/ or emotional trauma/ or post-traumatic stress/ or traumatic neurosis/	40889	Advanced
2	combat fatigue.mp.	40	Advanced
3	shell shock*.mp.	131	Advanced
4	or/1-3	40970	Advanced
5	hypothyroidism/ or hyperthyroidism/ or thyroid disorders/ or thyrotropin/ or thyroxine/	2219	Advanced
6	suicidal ideation/ or exp attempted suicide/ or exp suicide/	36011	Advanced

7	exp thyroid hormones/ or thyrotropin*.mp. or thyroxine*.mp. or levothyroxine*.mp. or t3.mp. or t4.mp. or hypothyro*.mp. or thyroid*.mp. [mp=title, abstract, heading word, table of contents, key concepts, original title, tests & measures]	7983	Advanced
8	thyroid gland/	239	Advanced
9	(4 or 6) and (5 or 7 or 8)	207	Advanced
10	limit 9 to all journals	192	

Scopus

(TITLE-ABS-KEY (suicid* OR ptsd OR ((combat OR war OR posttrauma* OR "post trauma*") W/3 (stress* OR psychologic* OR distress OR neurosis))) AND TITLE-ABS-KEY (thyroid* OR thyroxin* OR levothyroxin* OR hypothyroid* OR hyperthyroid* OR thyrotropin OR t4 OR t3 OR triiodothyronine)) AND (LIMIT-TO (DOCTYPE , "ar") OR LIMIT-TO (DOCTYPE , "re") OR LIMIT-TO (DOCTYPE , "le")) = 1432
