# Supplementary material for: Association of Thyroid Function with Suicidal Behavior: A Systematic Review and Meta-Analysis
Source: Medicina (Kaunas). 2021 Jul 15;57(7):714. doi: 10.3390/medicina57070714 (PMC8303342; doi:10.3390/medicina57070714)
Supplement: Supplementary file 1 [file medicina-57-00714-s001.zip › supplementary.pdf]

Table S1. Summary of risk of bias assessment for the included studies.

Study	Study type	**Selection** (Maximum: ☆☆☆☆)	**Comparability** (Maximum: ☆☆)	**Outcome** (Maximum: ☆☆☆)
Duval, 2010	Case-control	☆☆☆	☆☆	☆
Duval, 2017	Case-control	☆☆☆	-	☆☆
Jokinen, 2008	Case-control	☆☆☆☆	☆☆	☆☆
Jose, 2015	Case-control	☆☆☆☆	☆☆	-
Ozcan, 2016	Case-control	☆☆☆	☆☆	☆☆☆
Study	Study type	Selection (Maximum: ☆☆☆)	Comparability (Maximum: ☆☆)	Outcome (Maximum: ☆)
Baek, 2014	Cross-sectional	☆☆	☆☆	-
Berlin, 1999	Cross-sectional	☆☆☆	☆☆	☆
Butkute- Sliuoziene, 2018	Cross-sectional	☆☆	-	-
Khurshid, 2018	Cross-sectional	☆☆☆	☆☆	-
Kim, 2013	Cross-sectional	☆☆	☆☆	-
Maes, 1989	Cross-sectional	☆☆☆	☆☆	-
Pompili, 2012	Cross-sectional	☆☆☆	☆☆	☆
Peng, 2018	Cross-sectional	☆☆☆	-	☆

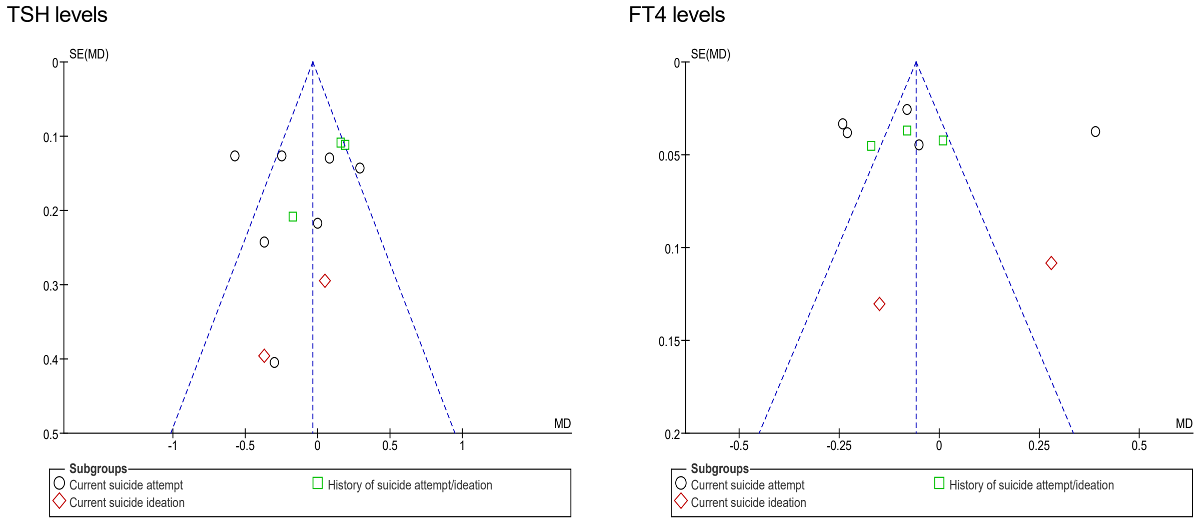

Figure S1. Funnel plots for publication bias analysis.

A) TSH levels

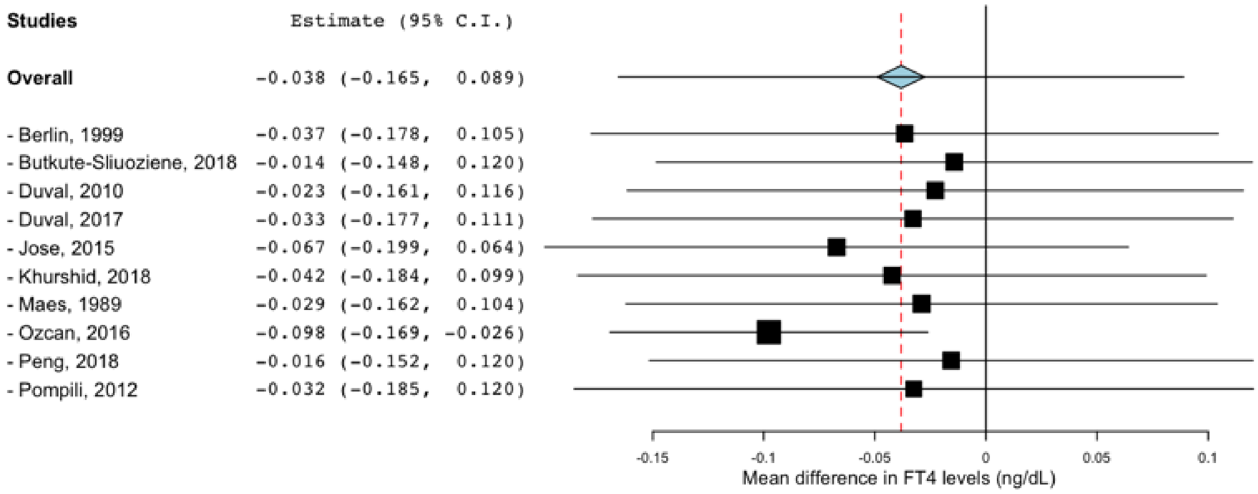

B) FT4 levels

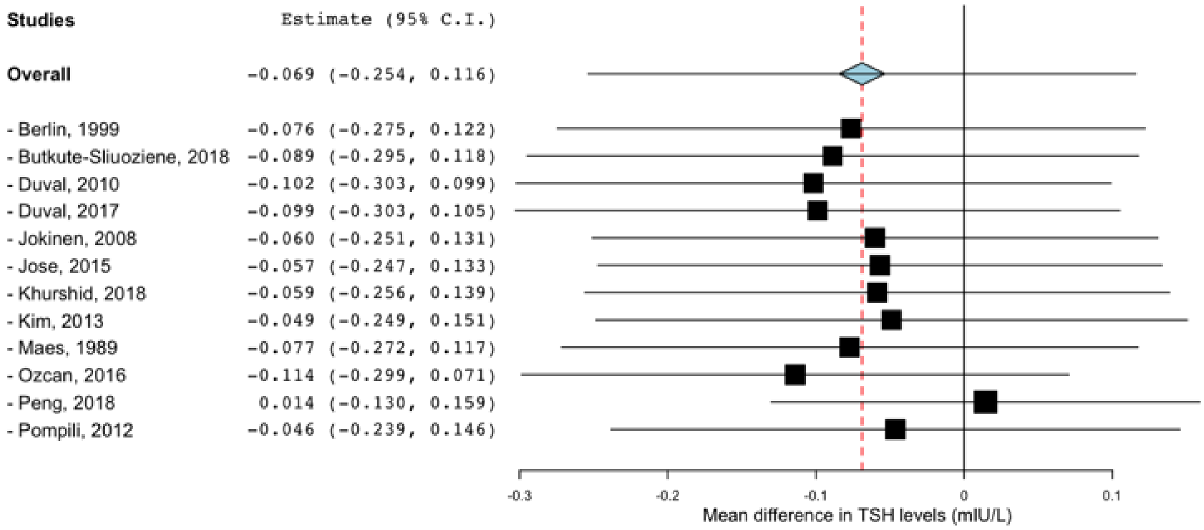

Figure S2. Leave-one-out sensitivity analysis of the studies included.
